# Clinicomicrobiological profile, visual outcome and mortality of culture-proven endogenous endophthalmitis in Taiwan

**DOI:** 10.1038/s41598-020-69251-0

**Published:** 2020-07-27

**Authors:** Ming-Chieh Hsieh, San-Ni Chen, Chieh-Yin Cheng, Kun-Hsien Li, Chih-Chun Chuang, Jian-Sheng Wu, Sheng-Ta Lee, Shin-Lin Chiu

**Affiliations:** 1 Department of Ophthalmology, Changhua Christian Hospital, No. 135, Nanxiao St., Changhua City, Changhua County 500 Taiwan, ROC; 2 Department of Optometry, Da-Yeh University, Changhua City, Taiwan, ROC; 3 School of Medicine, Chung-Shan Medical University, Taichung, Taiwan, ROC

**Keywords:** Bacterial infection, Fungal infection, Risk factors, Ocular motility disorders, Retinal diseases, Uveal diseases

## Abstract

This is a retrospective study in consecutive cases with cultured-proven endogenous endophthalmitis (EE) treated at the largest tertiary medical center in middle Taiwan in the past 10 years. 83 eyes of 70 patients were enrolled. The mean interval between systemic diseases to the diagnosis of EE was 8.84 ± 6.94 days. The mean initial visual acuity (VA) in the logarithm of minimal angle of resolution (logMAR) was 1.63 ± 0.87. Type 2 diabetes mellitus was the most common predisposing medical illness (N = 53, 63.86%). The most common infectious sources were intra-abdominal abscess (N = 36, 43.37%), and the second most reason was urinary tract infection. The causative pathogen was Gram-negative predominant (N = 64, 77.11%). After aggressive treatment, 34.94% of eyes regain useful vision, and only six eyes underwent enucleation or evisceration. The binary multivariate logistic regression model revealed that female gender (95% CI 1.002–19.036, p = 0.05, OR 4.37), initial VA logMAR (95% CI 0.089–0.550, p = 0.01, OR 0.22), and more intravitreal injections (95% CI 0.368–0.927, p = 0.023, OR 0.58) were independent risk factors influencing final outcomes. Based on the results mentioned above, early diagnosis is recommended to gain better outcomes. The mean interval between systemic diseases to the diagnosis of EE was 8.84 ± 6.94 days in our sample population, clinicians should maintain a higher index of suspicion during this period when encountering patients with bacteremia or fungemia.

## Introduction

Endogenous endophthalmitis (EE) is a vision-threatening disease that is often associated with several immunosuppressive conditions^1^. Because of the commonly poor prognosis, identifying the characteristics of EE and giving appropriate treatment are warranted.

To date, the management of EE continues to be a big challenge to ophthalmologists. Poor visual prognosis is found in most EE patients despite early interventions. Systemic antibiotics to control infectious progress is undoubtedly the mandatory treatment. In contrast, the roles of intravitreal antibiotics or steroid injection, subconjunctival antibiotics or steroid injection and early pars plana vitrectomy (PPV) remain unclear. Furthermore, the recommendations from the Endophthalmitis Vitrectomy Study may not be suitable for EE since the studied population was mainly based on patients with postoperative endophthalmitis, so-called exogenous endophthalmitis. Besides, to our best knowledge, no large-scale population-based study focusing on EE has been published to date, making it difficult to build any consensus.

The purpose of this study is to report on the characteristics of EE in patients who were referred to a tertiary medical center in Taiwan in the past 10 years. The systemic and ocular features, causative microorganisms, prognostic factors related to visual outcomes and mortality were identified. We also aimed to make recommendations on the management of endogenous endophthalmitis.

## Results

**Figure 1:**
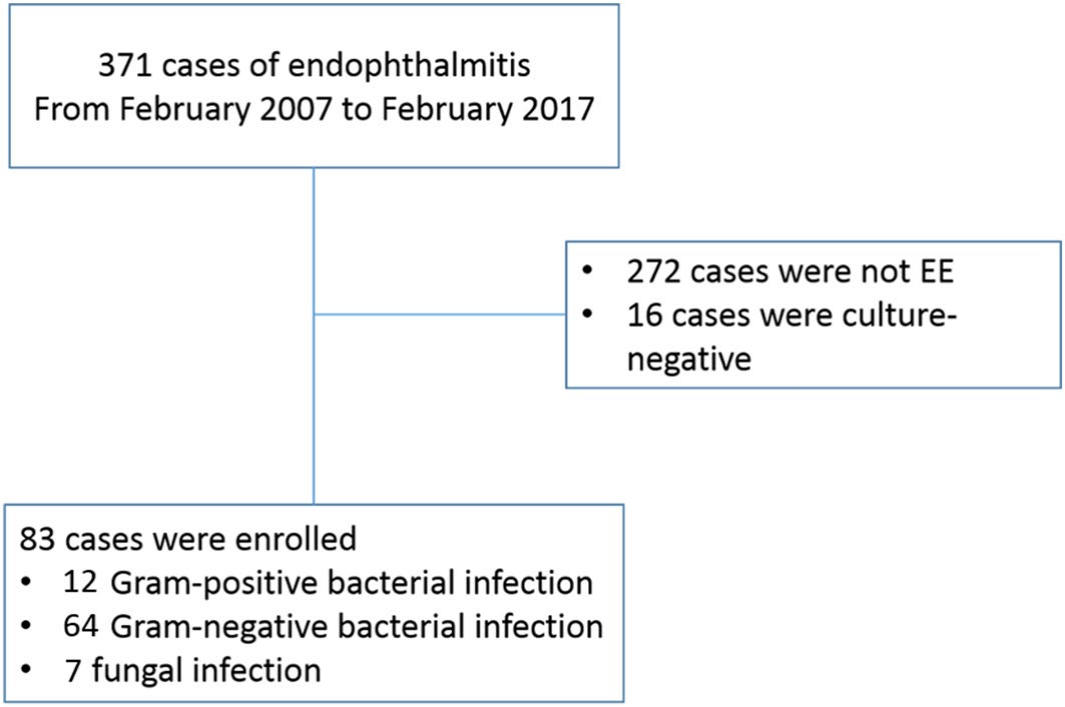
The flow-chart of data collection. *EE *endogenous endophthalmitis.

In the study period, a total of 371 episodes of endophthalmitis were managed at Changhua Christian Hospital, 288 cases were excluded for exogenous endophthalmitis. The remaining 70 patients with 83 incidences of culture proven EE were reviewed thoroughly. The flow-chart of data collection was shown in Fig. 1.

The mean age of the patients was 57.87 ± 17.22 years old. The mean interval between systemic illness to the diagnosis of EE was 8.84 ± 6.94 days. 56 infected eyes belonged to men and 27 belonged to women, respectively. Type 2 diabetes mellitus (DM) was the most common predisposing medical illness (N = 53, 63.86%) and the mean HbA1c of these diabetic patients was 9.05 ± 2.78%; only seven cases had no known history (8.43%) (Table 1).

**Table 1:** Baseline characteristics.

Involved eye OD:OS		39:44
Age		57.87 ± 17.22
Sex (male:female)		56:27
Interval between systemic disease to EE (days)		8.84 ± 6.94
Initial VA logMAR		1.63 ± 0.87

	N	Percentage
**Predisposing medical illness**		
Type 2 DM	53	63.86
Malignancy	11	13.25
Liver cirrhosis	7	8.43
ESRD under dialysis	7	8.43
Drug abuse	4	4.82
Old CVA	3	3.61
Glucose intolerance	1	1.20
HBV carrier	1	1.20
CAD	1	1.20
Thrombocytopenia	1	1.20
MV prolapse	1	1.20
No known history	7	8.43
**Source of infection**		
IAA	36	43.37
UTI	10	12.05
Pneumonia	4	4.82
CNS infection	2	2.41
Soft tissue infection	2	2.41
UC	1	1.20
IE	1	1.20
COM	1	1.20
IV tube	1	1.20
Not found	25	30.12
**Causative organism**		
Gram-positive	12	14.46
* S. aureus*	6	7.23
* Strep*. spp.	3	3.61
* Corynebacterium* spp.	1	1.20
MSSA	1	1.20
* Bacillus* spp.	1	1.20
Gram-negative	64	77.11
KP	44	53.01
PsA	6	7.23
* E. coli*	9	10.84
* A. baumannii*	2	2.40
Pm	1	1.20
* Samonella* spp.	1	1.20
* H. influenzae*	1	1.20
Fungus	7	8.43
* Candida albicans*	6	7.23
* Fusarium* spp.	1	1.20

*M*, male; *F*, female; *EE*, endogenous endophthalmitis; *VA logMAR*, visual acuity in logarithm of minimal angle of resolution; *DM*, diabetes mellitus; *HBV*, hepatitis B virus; *ESRD*, end stage renal disease; *CVA*, cerebrovascular accident; *MV*, mitral valve; *IAA*, intra-abdominal abscess; *UTI*, urinary tract infection; *UC*, ulcerative colitis; *IE*, infective endocarditis; *COM*, chronic otitis media; *IV*, intravenous; *S. aureus*, *Staphylococcus aureus*; *Strep* spp., *Streptococcus species*; *MSSA*, *Methicillin-sensitive Staphylococcus aureus*; *KP*, *Klebsiella pneumoniae*; *PsA*, *Pseudomonas aeruginosa*; *E. coli*, *Escherichia coli*; *A. baumannii*, *Acinetobacter baumannii*; *Pm*, *Proteus mirabilis*.

The most frequent infectious sources were intra-abdominal abscesses (N = 36, 43.37%), with 34 out of 36 being liver abscesses; the second most common cause was urinary tract infection (UTI, N = 10, 12.05%). The mean age of the cases caused by liver abscess was 56.85 ± 15.45, which showed no statistical difference (p = 0.63) comparing with the others; however, the mean age of the UTI cases was older, and was marginally significant when comparing with the others (67.30 ± 13.89, p = 0.05, Mann–whitney U test). Besides, a female predominance was noticed in the cases of UTI, and showed statistical difference comparing with other cases (p = 0.01, Fisher’s Exact test).

The causative pathogen was Gram-negative predominant (N = 64, 77.11%), as shown in Table 1. The initial visual acuity (VA) in the logarithm of minimal angle of resolution (logMAR) ranged from no light perception (NLP) to 0.15 and the mean VA logMAR was 1.63 ± 0.87. The initial VA logMAR showed no differences between liver abscesses and UTI cases (liver abscess: UTI = 1.51 ± 0.86: 1.49 ± 0.83, p = 0.22, Mann–Whitney U test). Red eye and pain were the most common complaints. Three of the affected eyes were asymptomatic. Cells in the anterior chamber, hypopyon and blurred or dirty vitreous were the most frequently described ocular signs.

Patients were managed differently depending on the situation. Except for seven cases, all of them received intravitreal injection (IVI) immediately in addition to systemic anti-microbial agents. The combination of teicoplanin and ceftazidime was the most preferred choice and the mean number of injections was 3.03 ± 2.27 times. Subconjunctival antibiotics injections were prescribed in 20 cases with a mean number of 4.15 ± 3.96 times. Only three cases were additionally managed by subconjunctival steroid injection and none got intravitreal steroid. PPV was performed in 39 eyes with a mean interval to operation time of 5.33 ± 7.48 days in these cases. In fact, 19 of them had PPV within 2 days after the diagnosis of EE was made. The reasons for postponed surgery were mostly related to the patients’ poor general conditions or their personal considerations. More than one surgery during follow-up was done in 12 patients, mainly due to rhegmatogenous retinal detachment after EE. On the other hand, six eyes got enucleation (N = 3) or evisceration (N = 3) during the study period.

The mean follow-up duration in this study was 330.17 ± 574.19 days. The mortality rate related to systemic illnesses was 10.00% (nine eyes of seven patients). The concerned outcomes were analyzed separately below.

### Outcome of interests: final visual acuity

**Figure 2:**
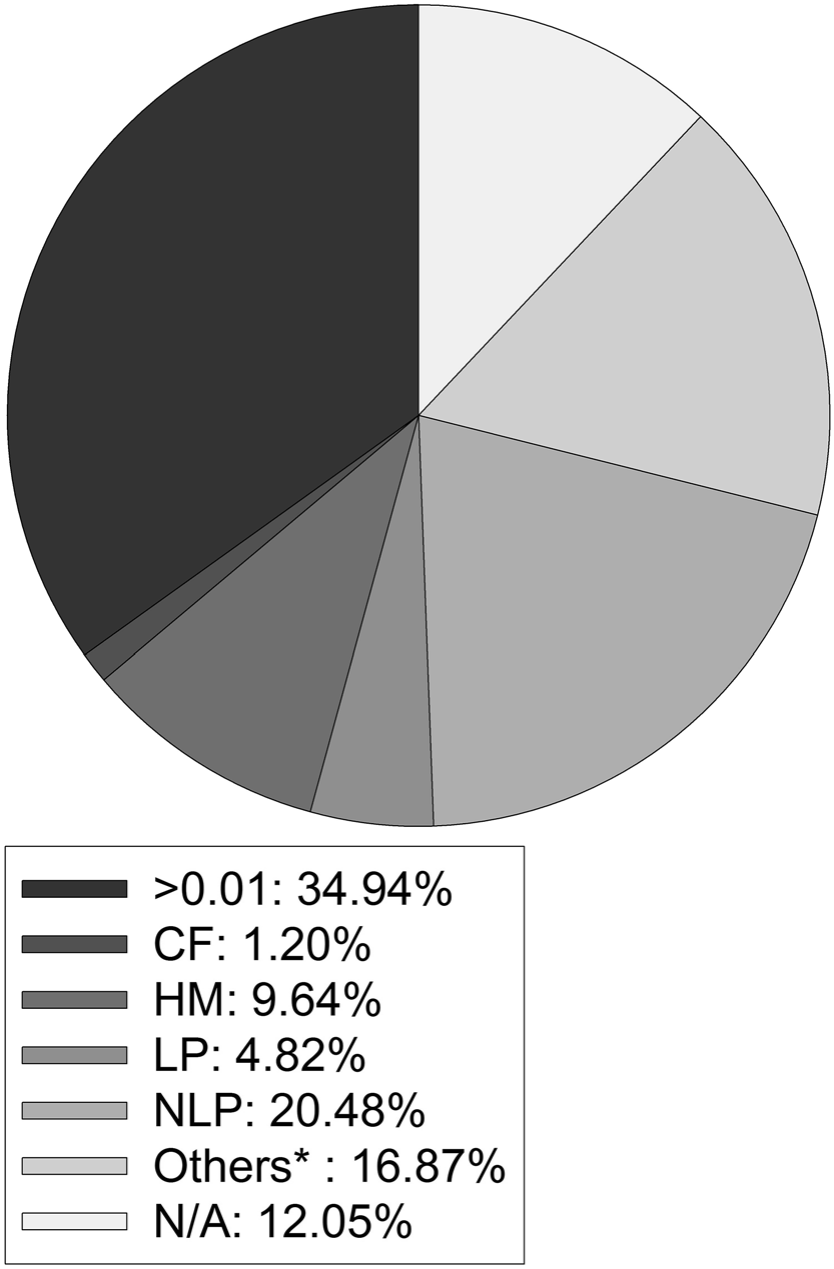
Final visual acuity during the follow-up period. *> 0.01* Snellen VA > 0.01, *CF* counting finger, *HM* hand motion, *LP* light perception, *NLP* no light perception, *N/A* data not applicable. Others*: including enucleation/evisceration and mortality cases.

The mean final VA logMAR of the 83 eyes was 1.77 ± 1.11, ranging from no light perception to Snellen VA 0.8. Among them, 34.94% of the cases regained vision better than counting finger, 7.23% of the cases underwent evisceration or enucleation; details were shown in Fig. 2. Although the paired-T test showed no statistical difference between the initial and final VA logMAR among all sample population (p = 0.19), after excluding cases caused by liver abscess, the Wilcoxon test disclosed a trend of better outcomes (initial VA logMAR:final VA logMAR = 1.71 ± 0.88:1.51 ± 1.08, p = 0.22). We analyzed the final VA of liver abscess and UTI cases seperately: cases of liver abscess demonstrated a significantly worse outcomes (initial VA logMAR:final VA logMAR = 1.51 ± 0.86:1.88 ± 1.23, p = 0.02, Wilcoxon test), wherars cases of UTI showed a trend of better outcomes (initial VA logMAR:final VA logMAR = 1.71 ± 0.95:1.36 ± 1.19, p = 0.53, Wilcoxon test).

An analysis was performed to compare bacterial and fungal EE. However, there was no difference between bacterial EE and fungal EE regarding the baseline characteristics, procedures they received, initial and final VA, and the rate of mortality. There was also no difference regarding baseline characteristics, procedures, initial and final VA logMAR, and the rate of mortality between Gram-positive and Gram-negative EE.

On the other hand, comparing with the unilateral cases, there was no male predominance in the bilateral cases (p = 0.04, Fisher’s exact test). Besides, the initial VA logMAR (bilateral: unilateral = 1.04 ± 0.76: 1.88 ± 0.79, p < 0.01, Mann–Whitney U test) and the final VA logMAR (1.35 ± 1.02: 1.96 ± 1.11, p = 0.03, Mann–Whitney U test) were significantly better in the bilateral involved cases. But the changes of VA logMAR at the end of their follow-up period showed no difference between the bilateral and unilateral cases (p = 0.48, Mann–Whitney U test). The interval between systemic illness and the diagnosis of EE was shorter in the bilateral cases (bilateral:unilateral = 8.15 ± 6.36:8.31 ± 7.47, p = 0.55, Mann–Whitney U test), though it did not reach statistical difference. Other factors including age, DM, procedures they received, follow-up period, and the rate of mortality remained no difference between the bilateral and unilateral cases.

The results of univariable and multivariable analysis of the effect of basic characteristics, causative pathogen and treatment strategy on visual outcome were shown in Table 2. Female gender, better initial VA logMAR, and having DM were positive factors predictive of regaining useful vision (counting finger or better), whereas having more intravitreal antibiotics injections was a negative factor, which were all statistically significant. Though Gram-negative EE had a tendency of worse outcome, it showed no statistically significance in both univariate and multivariate analysis. Besides, having PPV did not significantly regain vision.

**Table 2:** Analysis of vision better than counting finger in relation to basic characteristics, causative pathogen and procedures.

	Odds ratio (95% CI, p)	
	Univariable analysis	Multivariable analysis
Female	6.154 (0.091–0.939, p = 0.002)	4.367 (1.002–19.036, p = 0.05)
Without diabetes	0.292 (0.091–0.939, p = 0.039)	0.322 (0.075–1.389, p = 0.129)
Initial VA logMAR	0.261 (0.122–0.5610, p = 0.01)	0.221 (0.089–0.550, p = 0.01)
IVI	0.651 (0.475–0.891, p = 0.07)	0.584 (0.368–0.927, p = 0.023)

*VA logMAR* visual acuity in logarithm of minimal angle of resolution, *IVI* intravitreal injection

### Outcome of interests: evisceration and enucleation

During the study period, six cases of five patients received evisceration or enucleation. The mean age of the patients was 62.40 ± 24.64 years old. Two of them had type 2 DM and one had acute lymphocytic leukemia; the remaining had no known history. One of them had no VA data due to unclear consciousnesses; therefore, the following analysis only accounted for the other five cases.

The mean initial VA logMAR was 2.52 ± 0.41. The interval between the diagnosis and evisceration/enucleation was 4.50 ± 2.60 days. Eyes were mostly infected by Gram-negative bacteria (Gram-positive:negative = 1:4), whereas none of them had fungal infection. Among them, two cases were from *Klebsiella pneumoniae* (KP) related liver abscess. Details of the causative pathogen and the procedures received were listed in Table 3.

**Table 3:** Characteristics of the enucleation/evisceration cases (N = 6).

	N	Percentage
**Causative organisms**		
Fungus	0	0.00
Gram-positive	1	16.67
* Bacillus* spp.	1	16.67
Gram-negative	5	83.33
KP	3	50.00
PsA	1	16.67
* E. coli*	1	16.67
**Received procedures**		
IVI	4	66.67
SCI	2	33.33
PPV	3	50.00

*KP*, *Klebsiella pneumoniae*; *PsA*, *Pseudomonas aeruginosa*; *E. coli*, *Escherichia coli*; *IVI*, intravitreal injection; *SCI*, subconjunctival injection; *PPV*, pars plana vitrectomy.

Comparing with eyes who did not undergo evisceration/enucleation, poorer initial VA logMAR (p = 0.04, Mann–Whitney U test) has higher risks of having these kinds of debulking surgery. Other factors regarding sex, involved eye, causative organism, numbers of IVI, having DM or not, and having PPV or not remain no significant difference among these two groups.

### Outcome of interests: mortality

Nine cases of seven patients expired during their hospitalization. The mean age was 54.71 ± 16.04 years old, with a male predominance (men:women = 8:1). Five of them had DM with a mean HbA1c of 7.35 ± 1.52; four of them had malignancy. Three cases were unconsciousness at their first arrival at the hospital, the onset of systemic illness was unable to be determined. The interval between systemic illness to the diagnosis of EE for the other cases ranged from 2 to 30 days, with a mean of 7.83 ± 10.06. Most of them had no VA records because of unclear consciousness during the ocular examination. The details of causative pathogen were shown in Table 4.

**Table 4:** Causative pathogen of the expired cases.

	N	Percentage
**Causative organism**		
Fungus	0	0.00
Gram-positive	3	33.33
MSSA	1	11.11
* Strep*. spp.	1	11.11
* Bacillus* spp.	1	11.11
Gram-negative	6	66.67
KP	4	44.44
PsA	2	22.22

*MSSA*, *Methicillin-sensitive Staphylococcus aureus*; *Strep spp.*, *Streptococcus species; KP*, *klebsiella pneumoniae; PsA*, *Pseudomonas aeruginosa*.

## Discussion

EE is typically caused by hematogenous spreading of an actively infectious source^1^. The microorganisms in blood flow through blood-retinal barrier and induce serious intraocular infection and inflammation, which eventually lead to a poor prognosis. Although EE only accounts for 2–6% of endophthalmitis, it is still of great challenge to ophthalmologists because of the occult nature of the infection and the complicated co-morbid systemic disease conditions^1^. However, most publications are small case reports or series on this subject. Lack of large population-based studies make it difficult to build a consensus.

In the current study, the enrolled patients’ basic characteristics were collected. A significant male predominance was noted. The result was similar to the previous study^3^. However, if we analyzed the causes of EE separately, a female predominance was found in UTI cases (p = 0.01, Fisher’s Exact test). Since female sex is a risk factor for UTI, it is not surprising that EE from UTI is predominantly noted in female sex^4^.

In the meanwhile, the mean age was 57.87 ± 17.22, and the median was 58 years old, insinuating a higher frequency in the elderly. However, the mean patient age ranged from 35 to 68.5 years old in the published English literature^1,5^. The difference between our study and other reports is probably due to liver abscess being the leading factor in the enrolled cases (42.17%). The mean age of liver abscess cases was 56.85 ± 15.45 in our study, and the mean age of liver abscess in Taiwan is around 60-year-old^6^. On the other hand, we found out that the mean age of cases caused by UTI, the second most common cause, was older than the others and showed a marginal significance in our sample population (p = 0.05). This may be explained by that UTI are more common among aged women, with an annual incidence of 6–16% in women over the age of 65 and 20% in women over the age of 80 years of age^4^.

To be clear, 53.01% (N = 44) cases had KP infection in the present study, and 40.96% EE were caused by liver abscess; whereas only 14.46% EE were induced by Gram-positive bacteria. The close relation between EE and liver abscess were also noticed in the previous case series, in which the sample population were all Asians^2,7,8^. Besides, 63.86% (N = 53) cases in our study had DM. This finding is consistent with previously published data, that DM is the leading systemic comorbidity of EE^2,8,9^. In addition, DM is also the major underlying systemic risk factor for KP related liver abscesses^6,8,10^. The reason for this association is believed to involve both immunosuppression as well as a breakdown of the blood-retinal barrier in poorly controlled diabetics patients^8,9,11^. On the other hand, only 8.43% (N = 7) cases were fungus-related, which was very different from that in the Western World^3^. It is possibly related to the low incidence of intravenous drug use (IVDU) in our sample population (4.82%), while IVDU is believed to be the major cause of fungal EE^9,11^.

The mean interval between systemic illnesses to the diagnosis of EE was 8.84 ± 6.94 days. This is the first data which made note of the dangerous period after the onset of infection in Taiwanese. Muda et al. recently conducted a study showing that 37.6% of EE cases in Malaysia were diagnosed more than 1 month after the initiation of systemic illness^12^. This difference is possibly because of the high accessibility for patients seek for ophthalmic exams in our hospital, that Internists routinely consult Ophthalmologists for patients with bacteremia to rule out EE. Thus, appropriate diagnosis and treatment could be given earlier.

The initial VA logMAR was 1.63 ± 0.87. The most commonly found symptoms and signs were red eyes, eye pain, blurred vision, steamy cornea, hypopyon, and dirty vitreous, which were similar to other studies^12^. Only three cases had no identified symptoms before having an ocular exam.

The role of intravitreal injection and PPV remain controversial. Greenwald et al.^13^ strongly recommended withholding intraocular antibiotics in favor of more conservative therapy. They also found no compelling evidence that vitrectomy improved outcome, because none of the posterior diffuse EE in their series recovered useful vision, regardless of management. Reviewing the literature, the overall rate of vision recovery surpassing counting finger was around 22.64–34% in different literature; the main key factor for better outcomes was having better initial visual acuity^3,7^.

In our sample population, a more aggressively treatment strategy was chosen. A prompt empiric intravitreal injection was given in addition to systemic anti-microbial agents at the time EE was diagnosed. Despite the fact that in many cases, the causative pathogen was already known by blood culture, usually the choice of anti-microbial agent was either the combination of teicoplanin and ceftazidime or vancomycin and ceftazidime. We figured that it was based on the evidence that KP-related liver abscess has been the leading factor in EE among Taiwanese^7,14^. Besides, nearly half of the patients underwent PPV in the mean of 5.33 days. Though the role and timing of PPV remain controversial, PPV has its advantages of evacuating subretinal abscesses and securely obtaining adequate specimens for further investigation^9^. Some authors suggested that early PPV should be considered in whom anterior inflammation did not respond well to IVI^12^. However, although the time to operation in our study was shorter than previous studies^9,12^, we did not find significant influences in final VA.

To be clear, during this 10-year study period, after having several aggressively treatment as mentioned above, only six eyes (7.23%) underwent enucleation or evisceration and seven patients expired due to severe infectious condition. Our results were better than the published literature^3,7,9^. Also, after excluding cases of liver abscess, a trend of better outcomes was found (intial VA:final VA logMAR = 1.71 ± 0.88:1.51 ± 1.08, p = 0.22, Wilcoxon test).

On the other hand, in the present study, the initial and final VA were statistically better in the bilateral involved cases. But the amount of VA changes at the end of the follow-up period remained no difference between the two groups after treatment. The better final VA in the bilateral cases may be related to the better initial VA. Li et al., previously disclosed that comparing with unilateral cases, bilateral EE had better final VA, and the interval between decreased VA and ocular treatment was 1.5 days shorter in bilateral cases^8^. In our sample population, we did not document the interval between decreased VA and ocular treatment, but the interval between systemic illness and the diagnosis of EE was also shorter in the bilateral cases. However, the difference did not reach a statistically significant. Other factors also remained no difference between bilateral and unilateral EE. The precise mechanism for the better initial and final VA in bilateral cases remains unclear.

Univariate and multivariate analysis were performed to find out predictive factors influencing final visual outcomes. Female gender (95% CI 1.002–19.036, p = 0.05) was a protective factor, whereas having worse initial VA logMAR (95% CI 0.089–0.550, p = 0.01), and, surprisingly, having more IVI (95% CI 0.368–0.927, p = 0.023), were risk factors for not regaining vision to counting finger or more, statistically significant in both univariate and multivariate analyses. We hypothesize that it was because cases with poor responses to injections received more frequent treatment. Therefore, having more injections seemed to be a risk factor for poorer outcome. Although Gram-negative bacteria seemed to cause worse outcomes because of its’ capsule and endotoxin^5^, they did not seem to result in worsened outcomes our study. This lack of statistical significance in the association between visual outcome and type of causative pathogen in the present study may be attributed to the small sample size involved. Meanwhile, having DM was more likely to result in better outcomes in univariate analysis, but it did not reach statistically significance in multivariate analysis (95% CI 0.075–1.389, p = 0.129). Nishida et al. previously conducted an 11-year retrospective study of endogenous bacterial endophthalmitis^5^, which also reported significantly better outcomes in diabetic patients in their series. One possible reason is that in patients without DM, EE may be caused by different pathogens, including more invasive micro-organisms, therefore resulting in worse outcomes^5,15^. However, the definite answer remains unclear. Further studies are warranted for the pathogenesis of EE in diabetic cases.

The detailed data of the eyes which underwent enucleation/evisceration or patients who expired due to systemic disease were also documented. Among them, the top reason for EE was still KP-related liver abscess. The initial visual acuity was 2.52 ± 0.41 in the former, which was significantly worse than eyes not having to undergo enucleation/evisceration (p = 0.04). Whereas other factors regarding sex, age, diabetes status, IVI numbers, and having PPV or not remain no difference between the former and the later. Despite prompt and aggressive treatment, the former was still enucleated/eviscerated around 1 week later. This finding was consistent with previous reports that the worse the initial VA it is, the poorer outcomes it may have^2^. On the other hand, examination and treatment were hard to be given in the patients who expired due to systemic illness at the end of follow-up period because of severe infectious condition, therefore there were not enough data to be analyzed^12^.

The strength of our study is that the study period was 10 years, which made the sample population large enough for statistical analysis. But there are still several limitations. First, this is a retrospective study, and the data were collected from a single medical center. Besides, this is real-world practice, and the managements were based on the physician’s and patients’ discretion without following a definite protocol. And there were no control groups for outcomes comparing different treatment strategies. Future prospective studies are necessary to further clarify the treatment strategy in the management of EE.

## Conclusion

The top reason of EE in the present study was KP-related liver abscess, and the second reason was UTI. In contrast with previous studies, our sample population showed better visual outcomes. We also found out cases of UTI was older, had a female predominance, and had a trend of better outcome when comparing with the others.

The prognosis in our study depend on the initial VA while the diagnosis was made, female gender, and the numbers of IVI. Early diagnosis is recommended to gain better outcomes. Giving the mean interval between systemic illness to EE was 8.84 ± 6.94 days in our sample population, a higher index of suspicion should be maintained by clinicians during this period when encountering patients with bacteremia or fungemia.

## Methods

This is a retrospective, interventional, comparative study in consecutive cases with evidence of cultured-proven endogenous endophthalmitis treated at Changhua Christian Hospital, the largest tertiary medical center in middle Taiwan, from February 2007 to February 2017. The study was approved by the Institutional Review Board of Changhua Christian Hospital and was conducted in accordance with the tenets of the Declaration of Helsinki.

### Patient recruitment

Informed consent was obtained from all patients or their family. All cases with the diagnosis of endophthalmitis (ICD-9 360.00; ICD-10 H44) were reviewed retrospectively. The diagnosis of EE was defined as the presence of iritis and vitritis on ophthalmic examination and evidence of constitutional systemic infection. Patients with negative culture results, documented recent ocular trauma, corneal ulcer, ocular surgery in half a year or any other intraocular inflammation were excluded.

Systemic microbiological analysis such as complete blood counts, liver and renal function tests, culture from peripheral blood, HbA1c, and other specific tests and imaging were collected. Thorough ophthalmologic examination including visual acuity, slit-lamp biomicroscopy, indirect ophthalmoscopy and ultrasonography, as well as any ocular interventions were documented.

### Outcomes of interest

The primary outcome of interest was the final VA at their last follow-up and the rate of evisceration and enucleation. The secondary outcome was the mortality rate owing to related systemic illnesses.

### Statistical analysis

VA was measured on Snellen’s charts and converted to the logMAR units for statistical analysis. Non-numerical visual acuities were represented as the following: counting fingers (CF) = 1.7 logMAR, hand movement (HM) = 2.0 logMAR, light perception (LP) = 2.3 logMAR, NLP = 3.0 logMAR^2^. Phthisis, evisceration and enucleation were also represented by 3.0 logMAR.

All statistical analysis was performed on SPSS 22.0 software (SPSS, Chicago, IL, USA). Mann–Whitney U test was performed for continuous variables. For categorical variables, χ^2^-test or Fisher’s exact test if any cell frequencies were less than five were performed. Factors influencing the final visual outcome were analyzed by logistic regression. A significance level of P < 0.05 was used.
